# Prescriptions

**Published:** 1896-07

**Authors:** 


BILIOUSNESS.

℞. Sodii sulphat.,
Potassii et sodii tart ..aa ℥j.
Infusi cascarillæ......... ℥viij.

M. Sig.: Two tablespoonfuls three times daily.—FOTHERGILL.

DIABETES MELLITUS.

℞. Lithii carbonat........gr. xxx.
Sodii arseniat.........gr. j.
Ext. gentianæ .......gr. xv.

M. Ft. massa et in pil. no. xx div. Sig.: One pill morning and evening.—VIGIER, *Annual Univ. Med. Sci.*

DIARRHŒA, CHILDREN.

℞. Magnesii sulphat.........ʒj.

Tinct. rhei ...... ........ʒj.

Syr. zingiberis.............ʒj.

Aquæ carui................ʒix.

M. Sig.: A teaspoonful three times daily, to a child one year old.—WEST.

DYSENTERY.

℞. Cupri sulphat............gr. ss.

Magnesii sulphat........℥j.

Acidi sulphurici dil ....ʒj.

Aquæ..................℥iv.

M. Sig.: A tablespoon every four hours. (*In acute form.*)—BARTHOLOW.

HÆMOPTYSIS.

℞. Ext. ergotæ fld.........℥j.
Olei gaultheriæ..........gtt. iv.
M. Sig.: A teaspoonful every hour at first; then every four to six hours.—RINGER.

HEART DISEASE.

℞. Ferri redacti,
Pulv. digitalis fol. (English),
Quiniæ sulphatis......aa ℈j.
Pulv. scillæ ..... .......gr. x.
M. Ft. massa et in pil. no. xx div. Sig.: A pill three or four times daily. (*In fatty heart, dilatation of cavities, and mitral regurgitation, with anœmia.*)—BARTHOLOW.

WORMS.

℞. Olei terebinthinæ,
Oleoresinæ filicis maris, aa ʒj.
Mucilag. acaciæ............℥ij.
M. Ft. emulsio.
Sig.: Day before treatment, a milk or thin soup diet, and one drachm of compound jalap-powder. The emulsion is taken the following morning, fasting, and a half-hour later a dose of castor oil. (*For tape worm.*)—F. A. A. SMITH, *Annual Univ. Med. Sci.*

ORCHITIS.

℞. Antimonii et potassii tart. gr. j.
Aquæ ...................℥viij.
M. Sig.: One or two teaspoonfuls every hour or two.—RINGER.

